# Neonatal Cardiac Mesenchymal Stromal Cells Promote Recovery of Infarcted Myocardium through CD44 Mediated FoxP3^+^ T-Regulatory Cells after Vascular Infusion

**DOI:** 10.1007/s12015-024-10750-2

**Published:** 2024-06-28

**Authors:** Progyaparamita Saha, Sameer Ahmad Guru, Zhi-Dong Ge, Lydia Simms, Ling Chen, Roberto Bolli, Sunjay Kaushal

**Affiliations:** 1 Department of Cardiovascular-Thoracic Surgery, Northwestern University Feinberg School of Medicine, Chicago, IL USA; 2 Department of Pediatrics, Ann & Robert H. Lurie Children’s Hospital, Chicago, IL USA; 3 Division of Cardiovascular Medicine and Institute of Molecular Cardiology, University of Louisville, Louisville, USA

**Keywords:** Myocardial infarction, Mesenchymal stomal cells, Intravenous injection, Anti inflammation, Treg cells

## Abstract

**Abstract:**

Intravenous infusion has been used as the method of cell delivery in many preclinical studies as well as in some early clinical trials. Among its advantages are broad distribution, ability to handle a large-volume infusion, and ease of access. Progenitor cells used in cell-based therapy act through their secretomes, rather than their ability to differentiate into lineage-specific cell type. Since not all progenitor cells have similar secretome potency, the innate abilities of the secretome of cells used in clinical trials will obviously dictate their effectiveness. We previously found that cardiac neonatal mesenchymal stromal cells (nMSCs) are more effective in repairing the infarcted myocardium compared to adult mesenchymal stromal cells (aMSCs) due to their robust secretome (Sharma et al *Circulation Research 120*:816–834, 2017). In this study, we explored the efficacy of intravenous (IV) delivery of nMSCs for myocardial recovery. Six-week-old male Brown Norway rats underwent acute MI by ligation of the left anterior descending artery, followed by IV infusion of cell dose 5 × 10^6^ nMSCs/rat body weight (kg) or saline on days 0 and 5. We found that cardiac parameters in the rodent ischemia model improved 1 month after nMSCs infusion, and the result is comparable with the intramyocardial injection of nMSCs. Tracking the infused cells in target organ revealed that their movement after IV delivery was mediated by the cell surface receptor CD44. Systemic injection of nMSCs stimulated immunomodulatory responses specifically by increasing FoxP3^+^ T-regulatory cell influenced anti-inflammatory macrophages (M2) in heart. These data demonstrate that nMSCs promote immunogenic tolerance via CD44-driven T-reg/M2 stimulation that helps nMSCs for longer viability in the injured myocardium for better functional recovery. Our data also demonstrate a rationale for a clinical trial of IV infusion of nMSCs to promote cardiac function improvement in the ischemic patients.

**Graphical Abstract:**

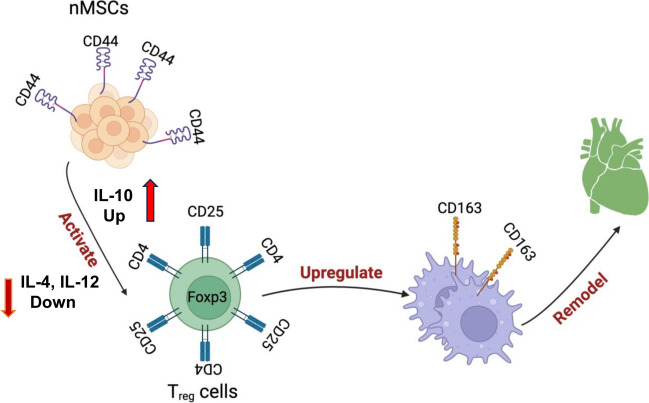

## Introduction

Progenitor cell therapy has garnered significant interest as a treatment strategy for a wide range of diseases over the last decade. Heart disease, peripheral vascular disease, bone disease, cancer, hepatic disease, and neurological disease have all been the focus of promising cell therapy breakthroughs. These cell-based therapies do not act through differentiation into lineage-specific cell types; rather, they act via the cells’ secretomes. The secretomes of different types of progenitor cells also differ in potency [1], which, in turn, will dictate the potency of cells used in clinical trials. Transplantation of bone marrow mononuclear cells (MNCs) in patients with acute myocardial infarction (MI) has not been effective [2] and has resulted in only minimal improvements in acute and chronic rodent models of MI [3]. These disappointing results underscore the importance of identifying effective reparative cells before embarking on clinical trials. Currently, invasive delivery routes limit clinical use of cell therapy. It is therefore critically important to determine whether intravenous (IV) delivery of cell therapy can provide serum secretome levels sufficient for promoting myocardial recovery. We previously found that due to their robust secretome, cardiac neonatal mesenchymal stromal cells (nMSCs) are more effective at repairing infarcted myocardium than adult mesenchymal stromal cells (aMSCs) [4]. Here we.

Tested the hypothesis that systemic infusion of nMSCs can improve the structure and function of infarcted rodent hearts. This article also established a possible mechanism of nMSCs’s mode of action to recover infarcted myocardium.

Innate and inflammatory immune responses play important role in ischemia-induced cardiac damage and repair processes by triggering a cascade of events with the aim of healing the injured tissue [5]. At the onset of injury, the neutrophils and monocytes/macrophages infiltrate in the tissue to remove necrotic debris. This is followed by resolution of inflammation, fibroblast activation, replacement fibrosis, and scar tissue formation [6]. Ischemia can cause a modulation of the immune response, which lead therapeutic benefit by expediting myocardial recovery [7]. Previous evidence suggests that MSCs modulate the immune response by inhibiting cytotoxic T cells and increasing proliferation of regulatory T cells (Tregs) [8]. Additionally, MSCs induce angiogenesis by polarizing macrophages toward the M2 phenotype [9]. Furthermore, CDCs have been shown to promote immune tolerance by activating programmed death ligand 1 (PD-L1), an immune checkpoint modulator that suppresses excessive immune activation [10]. These immune pathways triggered by transplanted cells have been recently questioned by some studies showing that transplanted cells failed to reduce inflammation, and the inflammation, which stimulated by transplanted dead cells a similar functional recovery was observed in a murine MI model [3, 11]. Thus, additional investigation is needed to further define the response of immune cells and their mechanism(s) for rejuvenate injured myocardium. This manuscript evidenced one of the mechanisms of nMSCs for recovering myocardium after IV infusion.

## Methods

### MI and Intravenous Cell Injection

To evaluate the in vivo immunogenicity and cardiac repair potential of nMSCs, nMSCs were infused intravenously vial tail vain to Brown Norway (BN) rats. Rats (6–8 weeks old) underwent left thoracotomy under isoflurane (2%) anesthesia and myocardial infarction (MI) was induced by permanent ligation of the left anterior descending (LAD) coronary artery using sutures. After confirming ischemia by visual inspection, the wound was closed. To determine the best dosage three different dosages like 1 M nMSCs / kg (group 1; N # 10), 5 M nMSCs / kg (group 2; N # 10), and 10 M nMSCs / kg (group 3; N # 10) suspended in 100 μL of vehicle (Iscove’s Modified Dulbecco’s Medium [IMDM]) or placebo control (group 4; N # 6) were intravenously injected to the BN rats via tail vain. Throughout the experiment male rats were used as hormonal changes in female rats may made them react differently to the same stimuli. To compare intramyocardial injection to intravenous injection 5 M nMSCs / kg (N # 10) were injected into the myocardium at 4 sites adjacent to the infarct region to MI rats. For in vivo potency determination of nMSCs, CD44 gene expression was knock down by shCD44 RNA, then nMSCs control (5 M nMSCs / kg, N# 8), nMSCs^scramble^ control (5 M nMSCs / kg, N# 5) and nMSCs^shCD44^ (5 M nMSCs / kg, N# 13) were intravenously injected to three different MI rats groups respectively. Transthoracic echocardiography was performed at post-operative days 1, 7, and 28. To evaluate cardiac function, left ventricular ejection fraction (LVEF) and left ventricular fractional shortening (LVFS) were assessed. Data were calculated from 5 cardiac cycles according to generally accepted formulas [12]. The experimental procedures were approved by the Animal Care and Use Committee of Northwestern University and conformed to the Guide for the Care and Use of Laboratory Animals (IACUC protocol # IS00016391)**.**

### Immunoblotting

Cell lysate proteins (30 μg) from nMSCs and aMSCs were electrophoresed on Bis-Tris gels (NuPAGE) and transferred to nitrocellulose membranes (Life Technologies). Blots were blocked with 5% nonfat dry milk at room temperature for 1 hour and then incubated overnight at 4 °C with monoclonal primary antibodies to poly [ADP-ribose] polymerase 1 (PARP1; BD61095), myeloid cell leukemia-1 (MCL1; cat# 4572S), B cell lymphoma-extra-large (BCL-xL; cat# MA5–15142), phosphorylated Bcl2-associated agonist of cell death (p-Bad; cat# 9295S), B cell lymphoma 2 (BCL2; cat# ab59348), CD44 (cat # 12–0441-82) and glyceraldehyde 3-phosphate dehydrogenase (GAPDH; MAB374) at the concentrations indicated in the manufacturer’s protocol, followed by incubation with fluorochrome-conjugated secondary antibodies (Odyssey, cat #926–32,219, cat #926–322,214) at room temperature for 1 hour. Blots were imaged using an Odyssey LI-COR.

### Lentivirus Production and Transduction

Manipulation of gene expression was performed by lentiviral transduction of nMSCs. The lentivirus mCherry^+^ expression system was obtained from Origin (catalog #TL710232). The titer of each lentivirus preparation was calculated based on the amount of virus required to yield 50% mCherry^+^ cells following transduction of 100,000 MSCs. Cells were transduced in 12-well dishes with increasing amounts of lentivirus in media supplemented with 8 μg/mL polybrene (Sigma Aldrich #TR-1003). Three days after transduction, to calculate the virus titer, the percentage of mCherry^+^ cells in each well were determined by flow cytometry using the LSR Fortessa. Cells were finally transduced in T25 flasks with the required amount of virus using polybrene. After 3 days, mCherry^+^ cells were detected by fluorescence-activated cell sorting (FACS).

### Isolation of PBMCs for Detection of mCherry^+^ Human Cells

After LAD ligation, mCherry^+^ nMSCs were transfused into rats via the tail vein. Blood samples were taken at different timepoints for isolation of peripheral blood mononuclear cells (PBMCs) using the BioVision kit (cat# K541–30) kit according to a modified version of the manufacturer’s protocol. After determining the live cell count, rat PBMCs were incubated at 4 °C for 20 minutes with anti-mCherry antibody (Cat # M11240). The percentage of mCherry^+^ cells was determined by FACS.

### Histology

Tissues were processed as described previously [4]. Briefly, after collection of echocardiographic data, rat hearts were excised under anesthesia and perfused with 10% formalin (Sigma Aldrich #HT501128). Tissues were cryopreserved in 30% sucrose in 1× PBS and embedded in optimal cutting temperature compound (Fisher Scientific, TissueTek #NC1029572). Sections (7 μm) were cut with a commercial cryostat, stained with different antibodies according to the manufacturers’ instructions, and counterstained with 4′,6-diamidino-2-phenylindole (Sigma #F6057) together with other required stains such as mCherry/CD44. All images (>5/slide) were obtained with an EVOS microscope and quantified using Image J software.

### Flow Cytometry Analysis

Heart tissue from treated animals was harvested at day 5 post-MI, minced, and then digested by collagenase D (Roche) at 37 °C for 50 minutes on a rocking platform (180–200 rpm). After enzymatic digestion, the cell suspension was filtered through a 70-μm cell strainer (Fisher Scientific #22363548) and centrifuged at 500×g for 10 minutes. To lyse red blood cells, the cell pellet was incubated in ammonium-chloride-potassium lysing buffer (Gibco # A10492–01) at room temperature for 3–5 minutes, then washed with iced cold fluorescence activated cell sorter washing buffer (2.5% fetal bovine serum in PBS without calcium and magnesium). Cells were resuspended in the washing buffer, and samples were incubated with Fc-Block (anti-rat CD16/CD32, 0.5 μg per 1 million cells) before incubation with isotype controls or primary antibodies, according to the manufacturer’s instructions. Cells were then washed with washing buffer. Approximately 2 × 10^5^ events (cells) were analyzed by flow cytometry (BD-LSRFortessa) and populations gated as detailed below and analyzed by FlowJo software. The antibodies are described in Table 1. T cells and Tregs were first gated (FSC-A vs SSC-A) as lymphocytes. For total T cells, the CD3 cells were gated and further analyzed for CD4 and CD8. For Tregs, CD4 cells were gated, from this gate, CD25^+^ and Fox-P3^+^ double-positive cells were determined. For macrophages, CD45 cells were gated. From CD45-positive cells, CD68 (Total macrophages) were gated and further analyzed for CD163 for M2 macrophages [13].

**Table 1:** Flow Antibody List

Flow Antibody	Company name	Catalog number
CD16/CD32	BD Pharmingen	553142
CD3	BD Pharmingen (APC)	557030
CD4	Biolegend (PE/Cy7)	201516
CD8	BD Horozon (V450)	561614
CD25	Invitrogen (APC)	17–0390-82
FoxP3	Invitrogen (PE)	12–5773-82
CD68	Biorad (FITC)	MCA341A488
CD163	Novus biotechne (APC)	NBP2-39099AF647
CD45	BD Horozon (V450)	561587

### Statistical Analysis

Unpaired nonparametric tests with Mann-Whitney’s correction were performed to compare 2 groups. For comparisons between more than 2 groups, 1-way analysis of variance (ANOVA) with Tukey’s post hoc test was performed. Grouped echocardiography data were analyzed by 2-way ANOVA with Bonferroni correction. Continuous data were plotted on box-and-whisker plots, with the middle horizontal line representing the median and the upper and lower whiskers representing the maximum and minimum values of nonoutliers, respectively. The number of subjects is displayed under each box and whisker column. Extra dots represent outliers. Data were analyzed using GraphPad Prism 9 software. *P* values from .01–.05, .01–.001, and .001–.0001 or < .0001 were considered significant, ^*^ very significant, ^**^ and extremely significant, ^***/****^ respectively.

## Results

**Fig. 1:**
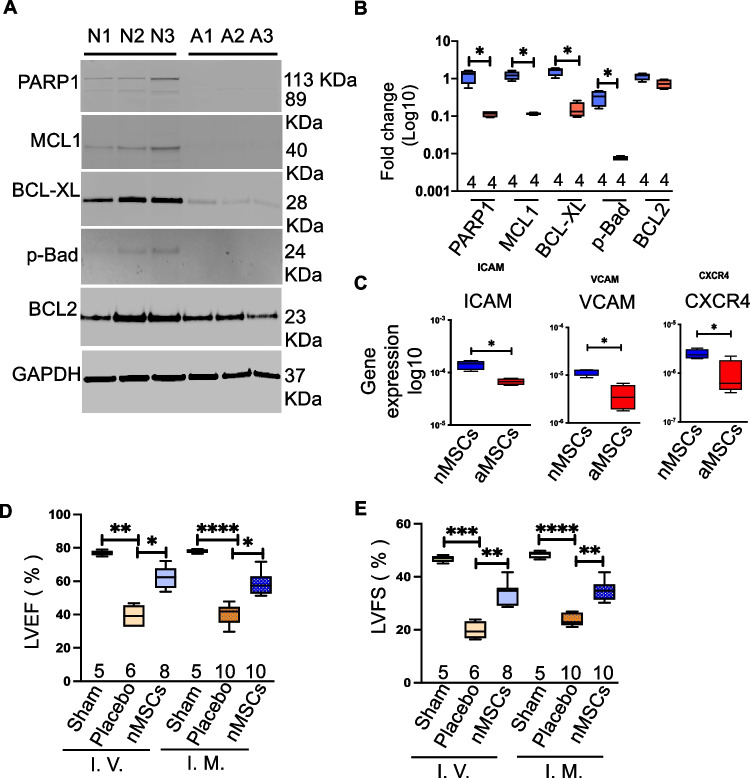
Intravenous infusion of nMSCs is comparable with intramyocardial injection of nMSCs. Immunoblots of anti-apoptotic proteins in neonatal and adult cells (**A**) and quantification (**B**). The expression of cell adhesion proteins like ICAM, VCAM and CXCR4 is determined by reat time PCR (**C**). Assessment of the cardiac parameters EF (**D**) and FS (**E**) by 2-dimensional M-mode echocardiography after IV infusion or IM injection of the cell dose (5 × 10^6^/kg). aMSCs = adult mesenchymal stem cells; BCL2 = B cell lymphoma 2; BCL-XL = B cell lymphoma-extra-large; Exo = exosomes; GAPDH = glyceraldehyde 3-phosphate dehydrogenase IM = intramyocardial; IV = intravenous; LVEF = left ventricular ejection fraction; LVFS = left ventricular fractional shortening; MCL1 = myeloid cell leukemia-1; nMSCs = neonatal mesenchymal stem cells; PARP1 = poly [ADP-ribose] polymerase 1; pBAD = phosphorylated Bcl2-associated agonist of cell death

We previously found that compared with aMSCs, nMSCs have superior cell characteristics based on growth properties, telomere shortening, and induction of senescence markers [4] with increasing passages. Here, we confirmed the presence of anti-apoptotic proteins in different nMSC or aMSC cell lines. Immunoblotting revealed high levels of expression of anti-apoptotic proteins, including PARP1, MCL-1, BCL-xL, and p-Bad in nMSCs but not in aMSCs (Fig. 1A, B), which might explain the functional superiority and sustainability of nMSCs in blood stream after cells injection. These data suggest that the intrinsic properties of nMSCs may enable them to resist cell death induced by reactive oxygen species and to better survive in a hostile, inflammatory environment. Cell adhesion molecules promote cell to cell and cell to extracellular matrix adhesion and thus help cells to migrate. Adhesion molecules like ICAM, VCAM and CXCR4 expression is significantly high in nMSCs compared to adult MSCs (Fig. 1C). To assess the in vivo functionality of the nMSCs, 6-week-old male Brown Norway rats underwent acute MI by LAD ligation, followed by IV infusion (via the tail vein on days 0 and 5) or intramyocardial (IM) injection of nMSCs (5 × 10^6^ cells/kg) or saline. Administration of nMSCs by either route resulted in significantly improved cardiac function by 4 weeks post-transplantation, as reflected by LVEF (Fig. 1D) and LVFS (Fig. 1E).

**Fig. 2:**
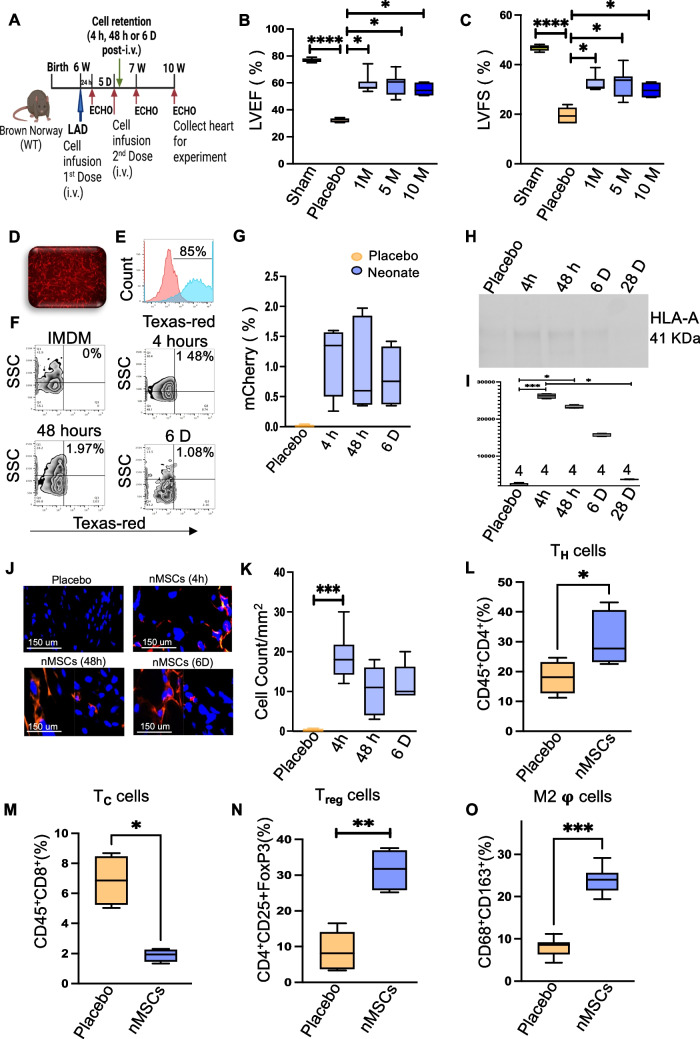
Detection of nMSCs in the system after intravenous infusion. (**A**) Experimental scheme for detection of nMSCs and timeline for cell retention studies using mCherry^+^ labeled nMSCs isolated from the right atrial appendage of neonatal human hearts. The cardiac parameters LVEF (**B**) and LVFS (**C**) were assessed by echocardiography after IV infusion of different doses of nMSCs (1 × 10^6^/kg, 5 × 10^6^/kg, 10 × 10^6^/kg). (**D**) Representative images of nMSCs transduced with a lentiviral vector to obtain a permanent mCherry^+^ cell line. (**E**) shows the % of transduced cells after Flow sorting. Quantitative flow cytometry analysis of mCherry fluorescence in the peripheral blood of post-MI Brown Norway rats at different timepoints after infusion with nMSCs (**F**) and quantification (**G**). (**H**) Representative Western blot for HLA-A detected in rat serum after tail vain injection of nMSCs at different time points and (**I**) shows the graphical representation of quantification of the protein blot. (**J**) Representative pictures of detection of mCherry leveled nMSCs in the injured myocardium at different time point by immunohistochemistry and (**K**) shows the quantification of staining. Immune cells were detected from cardiac single cell suspension by FACS at day 7 of nMSCs infusion. (**L**) shows CD4^+^ T cells (**M**) represents the CD8^+^ T cells, (**N**) displays the Treg cells and (**O**) exabits the status of anti-inflammatory M2 macrophage. Echo = echocardiography; MI = myocardial infarction; WT = wild type; other abbreviations as in Fig. 1

The nMSCs were transduced with mCherry lentivirus so that they could be tracked in the blood and organs at various timepoints after IV delivery, as outlined in Fig. 2A. For further investigation of vascular infusion of nMSCs, ischemic rodent model was infused with different nMSCs dosage to find out the appropriate dose of cells that can home and restore contractile function to the injured heart. Three different nMSC doses (1*10^6^/ kg, 5*10^6^/kg, and 10*10^6^/kg) were systemically infused into rodents. Compared with placebo, all 3 doses resulted in significantly higher LVEF (Fig. 2B) and LVFS (Fig. 2C). To assess nMSCs in the circulation, nMSCs were transfected with mCherry^+^ lentivirus, selected by puromycin treatment (Fig. 2D), with further confirmation by FACS (Fig. 2E). The mCherry^+^ nMSCs (5 M/kg) were systemically infused into ischemic rodents via the tail vain, and fluorescent cells from the peripheral blood were detected by flow cytometry at different timepoints (4 hours, 48 hours, and 6 days) post-infusion. Transplanted mCherry^+^ nMSCs accounted for approximately 1.5%–2% of circulating blood cells at different timepoints after tail vein infusion in the ischemic rodent model (Fig. 2F) and quantification is showed in 2G. Furthermore, at 4 h, 48 h and at 6 days post–IV injection, nMSCs were detected in the recipient serum by anti HLA-A antibody (Fig. 2H) and Fig. 2I showed the quantification of the presence of HLA-A antigen in earlier time points whereas none at 28 days in rat serum. The presence of mCherry^+^ nMSCs post-infusion in hearts was further evaluated by immunohistochemistry and quantified at 3 different timepoints (Fig. 2J, K). To determine the immunomodulatory effects of nMSCs in vivo, rats were systematically infused with nMSCs twice after MI (at day POD 0 and POD 5) with dose 5*10^6^/ kg and at day 7 the presence of T-regs, Helper T cell (CD4^+^), T-cells (CD8^+^), and M2 macrophages were evaluated in single-cell suspensions from the hearts with flow cytometric analysis. Infusion of nMSCs induced a significant increase of T_H_- cells (Fig. 2L), significant decrease of cytotoxic CD8^+^ cells (Fig. 2M) whereas CD4^+^/CD25^+^/FoxP3^+^ Tregs and CD68^+^/CD163^+^ M2 macrophages increased significantly (Fig. 2N & Fig. 2O) in the injured myocardium.

**Fig. 3:**
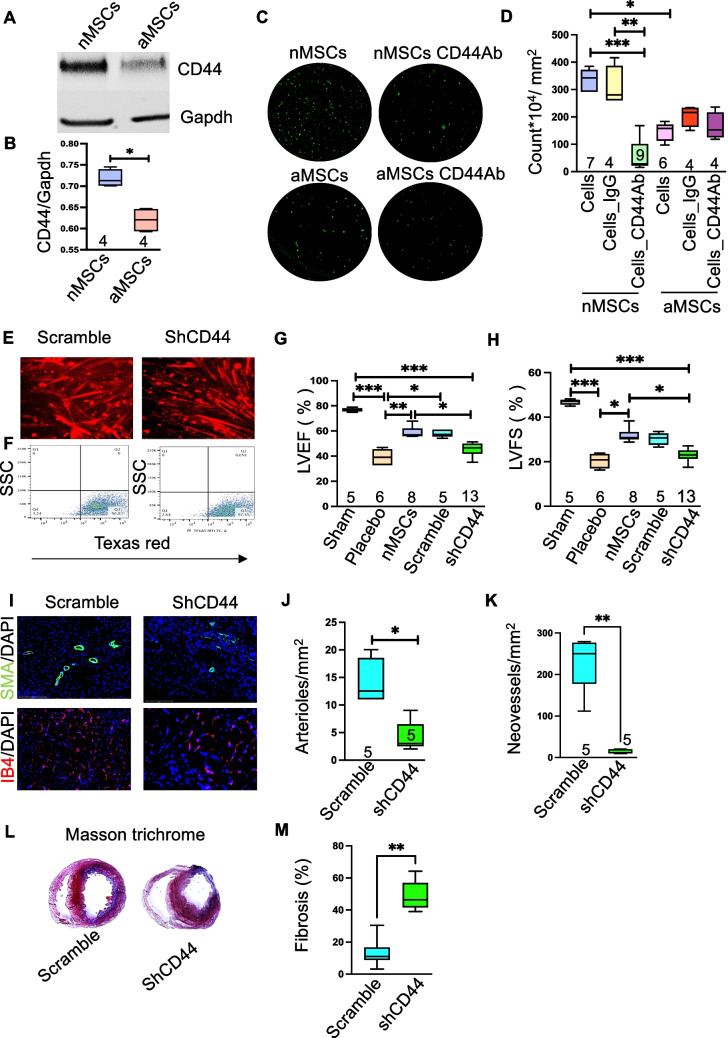
CD44 mediates movement of nMSCs to reach target organ after cell transfusion. Representatives immunoblot of basal expression levels of CD44 protein in nMSCs and MSCs (**A**) and quantification (**B**). (**C**) Representative pictures of trans well migration assay and (**D**) Quantification of the in vitro migration assays with the experimental group using an anti-CD44 antibody to block CD44 in aMSCs and nMSCs, with IgG used as a control. nMSCs were transduced with mCherry labeled lentiviral vector to perform the in vivo studies in rat MI model. (**E**) shows the representative pictures nMSCs^scramble^ (control) and nMSCs^shCD44^ after transduction. (**F**) represents the picture of sorted cells. Comparison of LVEF (**G**) and LVFS (**H**), after administration of nMSCs where CD44 is blocked by shCD44 (nMSCs^shCD44^) or the scramble control (nMSCs^scramble^), at the fourth week after LAD ligation. Myocardial sections were evaluated at 28 days post-transplantation for angiogenesis by co-staining for IB4 and SMA. Representative pictures are shown in (**I**). Quantifications are depicted for SMA (**J**) and IB4 in (**K**). Images (**L**) and percent scar size (**M**) from Masson trichrome staining. LAD = left anterior descending coronary artery; other abbreviations as in Fig. 1. SSC = side scatter; FSC = forward scatter; SMA = smooth muscle actin; IB4 = Isolectin B4

The transmembrane receptor CD44 was previously shown to facilitate glioblastoma cell migration [14] and may integrate adhesive and signaling activities to modulate the migratory processes. To determine the mechanism of nMSC movement after systemic infusion, we measured basal levels of CD44 by immunoblot analysis in nMSCs and aMSCs. Compared with aMSCs, nMSCs expressed significantly higher levels of CD44 protein in Fig. 3A and Fig. 3B showed its quantification. To determine whether nMSC migration is mediated by CD44, antibodies against CD44 was used to abrogate nMSC migration in transwell migration assays. In this assay, the migration of nMSCs was significantly decreased compared with that of aMSCs after anti-CD44 antibody was applied to the cells (Fig. 3C) and quantification of migrated nMSCs dyed with calcein is shown in Fig. 3D. To determine if a similar mechanism was responsible for nMSC migration in vivo, the nMSCs used in our in vivo rodent model of myocardial ischemia were transfected with an mCherry^+^ shCD44 lentivirus vector or with a scramble RNA control separately to obtain a CD44 knockdown and control nMSC lines, which were then infused (5*10^6^/kg) intravenously into the ischemic rodent model (Fig. 3E & F). Echocardiograms were obtained 4 weeks after IV infusion of nMSCs^shCD44^ or nMSCs^scramble^ (negative control) to determine if the rodents recovered myocardial function. We found that cardiac functions (EF and FS) were significantly lower in the rats infused with nMSCs^shCD44^ compared with the rats infused with the nMSCs^scramble^ or with nMSCs (Fig. 3G, H). Similarly, postmortem immunohistochemistry at 28 D with rat hearts showed significant decrease in arterioles and neo vessels formation (pictures in Fig. 3I, quantification Fig. 3J and Fig. 3K respectively). The extent of post-MI fibrosis after 28 days was determined by measuring the area of fibrosis (blue) relative to the total myocardial area (pink and blue) after Masson’s trichrome staining of heart sections in the groups (Fig. 3L). Fibrosis increased significantly in the group of rats which are infused with nMSCs^shCD44^ compared with scramble nMSCs (Fig. 3M).

**Fig. 4:**
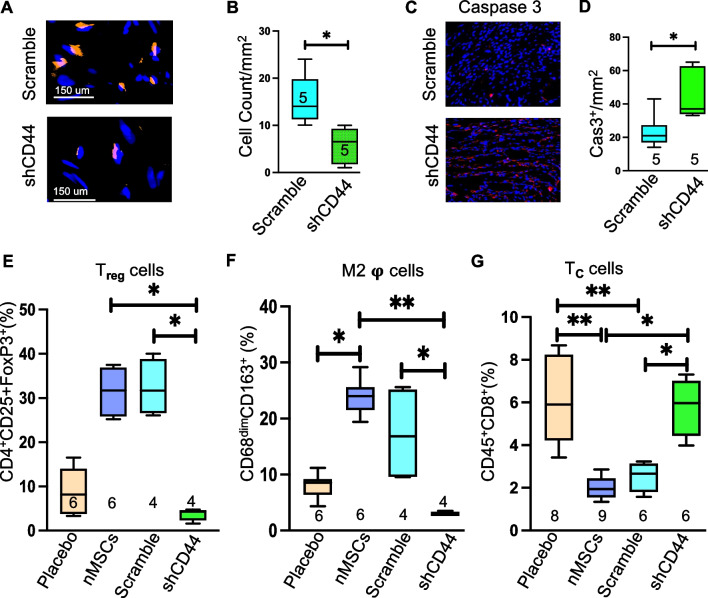
nMSCs promote recovery of infracted myocardium through immune modulation by CD44 mediated T-reg upregulation. Representative cell retention pictures at day 5 after vascular infusion of nMSCs^scramble^ and nMSCs^shCD44^ are shown in (**A**) and its quantification in (**B**). (**C**) represents the pictures of immune histochemistry of Caspase 3 and its quantification (**D**). Immune cells were detected from cardiac single cell suspension by FACS at day 7 of nMSCs^scramble^ and nMSCs^shCD44^ infusion. (**E**) displays the T_**reg**_ cells (**F**) represents the status of anti-inflammatory M2 macrophage (**G**) the CD8^+^ T_**C**_ cells. T_**reg**_ = T regulatory cells; 𝛗 = macrophages; T_**C**_ = cytotoxic T cells

Next, we confirmed that the migration of nMSCs from the site of intravenous infusion effects the immune modulatory behavior of the cells to the target organ. The pictures in Fig. 4A showed the poor engraftment of nMSCs^shCD44^ in injured myocardium compared to nMSCs^scramble^ and the quantification (Fig. 4B) showed significant negligible retention of nMSCs^shCD44^ after a week of cells infusion. Whereas significant increase of caspase 3 in the group nMSCs^shCD44^ (Fig. 4C and quantification Fig. 4D) further established the fact that the cells were dying in the injured myocardium. To evaluate the immune cells status rats were systematically infused with nMSCs^shCD44^/ nMSCs^scramble^ twice after MI (at day POD 0 and POD 5) with dose 5*10^6^/ kg and at day 7 the presence of T-regs, Helper T cell (CD4^+^), T-cells (CD8^+^), and M2 macrophages were evaluated in single-cell suspensions from the hearts with flow cytometric analysis. CD4^+^/CD25^+^/FoxP3^+^ Tregs (Fig. 4E) and CD68^+^/CD163^+^ M2 macrophages (Fig. 4F) were significantly decreased in the group nMSCs^shCD44^ compared to nMSCs^scramble^/nMSCs. Whereas the CD8^+^ T_C_ was significantly increased in nMSCs^shCD44^ compared to nMSCs^scramble^/nMSCs (Fig. 4G).

## Discussion

The repeated treatment concept for the patients depending on their disease situation led to a fundamental question that whether cells can be delivered intravenously to achieve the best treatment. So intravenous (IV) delivery of stomal cells must be interrogated deeply as IV results in only a minuscule percentage of cells engrafting in the heart. We had showed previously that cells stay almost 7 days in the myocardium after intramyocardial injection and their beneficial effects are mediated by release of various paracrine factors to reduce collagen deposition, inhibit apoptosis, augment angiogenesis, inhibit inflammation and suppress innate and adaptive immunity [4]. In this study we investigate whether the similar beneficial effects can be obtained by IV administration of cells and release of paracrine factors into systemic circulation, then this approach should obviate the need of intramyocardial injection of cells and will make cell therapy cheaper and safe. Our group proved previously that nMSCs overpowered adult derived stomal cells to recover ischemic myocardium through its robust secretome. Currently we investigated the important cell characteristics like cell survival, cell adhesion and cell migration properties of nMSCs, which help them to home back to target organ after systemic delivery. The antiapoptotic proteins (Bcl-xl, Mcl-1, PARP-1, bcl-1) inhibit both mitochondria-mediated and the death receptor-mediated pathways of apoptosis, here we demonstrated that nMSCs have significant basal level of anti-apoptotic protein compared to adult cells which provide them sustainability while intravenous delivery. The transmembrane receptor CD44, ICAM, VCAM was previously shown to facilitate cell migration [14] and may integrate adhesive and signaling activities to modulate the migratory processes. Musso et al. demonstrate earlier that ICAM, VCAM in intestinal fibroblast is responsible for the interaction with T cell and thus cause attenuation of gut inflammation in IBD. Similarly in the present study we showed that nMSCs are enriched with ICAM, VCAM which are involved in the control of T cell activation leading to immunosuppression, and nMSCs are also rich in CD44 proteins necessary for cell docking and migration after systemic infusion. A comparative study, of nMSCs IV delivery with intramyocardial delivery proved their efficacy to recover myocardial function after ischemia irrespective of their delivery route. We had published previously that intramyocardial injection of stomal cells release exosomes reflecting parent cell-specific surface markers into the recipient circulation. More recently we published that injection of nMSCs decrease the pro-inflammatory cytokine IL-4 and IL-12 and increase significantly anti-inflammatory cytokine IL-10 in the rodent serum. Our current study demonstrates the efficacious nMSCs dosage for intravenous cell therapy and most importantly detects the HLA-A specific exosomes in the recipient circulation. Detection of transplanted cells’ specific exosomes in the recipient serum establish the importance of paracrine mechanism. Moreover, the presence of exosomes in the serum highlights the importance of secretome potency of the cells to be used therapeutically. We and others have demonstrated the distribution of stomal cells in different organs quantitatively by PCR after systemic delivery. The novelty of our study here, is we observed significantly less cardiac improvement, cells retention in myocardium when nMSCs are devoid of CD44 transmembrane protein. Our study first time reported that cardiac-derived cell nMSCs, could promote LV functional recovery after IV injection through a CD44-driven mechanism. As we know immune cells play a critical role in ischemia-induced adverse cardiac remodeling. This process has 3 distinct phases: 1) an inflammatory phase, where cardiac cells necrosis triggers innate immune responses, promoting infiltration of the infarcted region by neutrophils and monocytes; 2) a proliferative phase, characterized by upregulation of T-regulatory cells and M2 macrophages involved in preliminary tissue stabilization by inducing repair processes such as angiogenesis; 3) a final phase is designated by the infarcted area being fibrotic, cardiac cells undergoing apoptosis, and diminished inflammatory response [15]. Modulation of immune responses during tissue remodeling is thought to be a therapeutic target for augmentation of tissue healing and repair in MI. So, in this study the immunomodulatory properties of nMSCs were observed under fully immunocompetent conditions. Our results suggests that intravenous infusion of nMSCs exert immunomodulatory effects by inhibiting the T cell proliferation, promoting Treg proliferation and most importantly enhancing the monocyte differentiation in to anti-inflammatory M2 macrophage phenotype. Our findings are consistent with previous reports suggesting a beneficial role for Tregs in cardiac repair of the infarcted myocardium [12]. Tregs inhibit CD4^+^ and CD8^+^ T cell proliferation and inhibit the secretion of interferon gamma [16]. Additionally, Tregs play an important role in polarization of macrophages toward the M2 phenotype [17], which, in turn, plays an important role in post-infarct tissue repair [18].

Systemic administration of nMSCs offers a minimally invasive route for cells delivery which is especially attractive for multiple dosing regimens. Moreover, the functional myocardial improvement effected by the nMSCs, coupled with superior paracrine effects, suggests that recent negative results with different cell treatment were due to the use of an ineffective cell type. The present data may provide a rationale for clinical trial of nMSCs to the ischemic patients in future.

## Data Availability

All the data related to this research work has been included in the manuscript. The experimental procedures and protocols are available from the corresponding author upon request.

## Abbreviation

IV: Intravenous
LAD: left anterior descending artery
MI: myocardial infarction
IM: Intramyocardial
nMSCs: neonatal mesenchymal stomal cells
